# Yeasts and Lactic Acid Bacteria for *Panettone* Production: An Assessment of Candidate Strains

**DOI:** 10.3390/microorganisms9051093

**Published:** 2021-05-19

**Authors:** Luciana De Vero, Giovanna Iosca, Salvatore La China, Fabio Licciardello, Maria Gullo, Andrea Pulvirenti

**Affiliations:** 1 Department of Life Sciences, University of Modena and Reggio Emilia, 42122 Reggio Emilia, Italy; luciana.devero@unimore.it (L.D.V.); giovanna.iosca@unimore.it (G.I.); salvatore.lachina@unimore.it (S.L.C.); fabio.licciardello@unimore.it (F.L.); maria.gullo@unimore.it (M.G.); 2 Interdepartmental Research Centre for the Improvement of Agri-Food Biological Resources (BIOGEST-SITEIA), University of Modena and Reggio Emilia, 42124 Reggio Emilia, Italy

**Keywords:** sourdoughs, starter culture, sequencing, volatile organic compounds, Panettone

## Abstract

The recovery of yeasts and lactic acid bacteria (LAB) involved in sourdough fermentation is the first step in the selection of starters with suitable technological aptitude and capable of producing desired aromas and/or aromatic precursors. In this work, two sourdoughs samples (MA and MB) and the derived doughs (samples A and B) were collected from a bakery during artisanal Panettone manufacture. Yeasts and bacteria were isolated at different fermentation steps on selective agar media. A total of 77 isolates were obtained and characterized. Representative strains of yeasts and LAB were identified by sequencing the D1/D2 domain of the 26S rRNA and the 16S rRNA genes, respectively. Moreover, the volatile organic compounds (VOCs) produced in the collected samples were detected and correlated to the species found in the same samples. The results highlighted the occurrence of *Kazachstania humilis* in both samples A and B, while *Saccharomyces cerevisiae* strains were detected only in samples B. Among LAB, *Fructilactobacillus sanfranciscensis* was the main species detected in both sourdoughs. Furthermore, strains belonging to the species *Lactiplantibacillus plantarum*, *Furfurilactobacillus rossiae*, *Lactobacillus parabuchneri*, *Leuconostoc citreum*, and *Leuconostoc mesenteroides* were assessed in the dough samples.

## 1. Introduction

Panettone is a traditional Italian baked cake produced from sourdough, also called mother dough (MD). Sourdough can be defined as a matrix of flour and water including yeasts and lactic acid bacteria (LAB) as main functional microorganisms [1,2].

Commonly, the type I sourdough is specifically used for the artisanal production of Panettone and other traditional Italian sweet baked products, such as Pandoro and Colomba [3,4,5]. This type of sourdough is characterized by daily refreshments, carried out every 4–16 h at 25–35 °C according to a back-slopping procedure, which consists of the addition of flour and water with an aliquot of previously fermented dough [6,7]. This practice maintains the microorganisms in an active state and promotes the selection of a stable and characteristic microflora, which is well adapted to the specific recipe and manufacturing procedure [8,9].

The main species occurring in type I sourdoughs are the LAB Fructilactobacillus sanfranciscensis (formerly Lactobacillus sanfranciscensis), Levilactobacillus brevis (formerly Lactobacillus brevis), and Lactiplantibacillus plantarum (formerly Lactobacillus plantarum) and the yeasts Saccharomyces cerevisiae, Kazachstania exigua, and Kazachstania humilis [7,10,11].

Technological parameters, such as temperature, flour composition, degree of dough hydration, and sodium chloride content significantly contribute to the selection of the microflora [12]. Moreover, the richness and diversity of yeasts in sourdoughs can differ considerably as they are influenced by many factors including tolerance to the organic acids produced by the LAB and the availability of carbon sources [12,13,14].

Generally, LAB activity causes the acidification of sourdough, while yeasts are mainly responsible for the dough leavening [4,6]. In addition, the metabolism of LAB and yeasts contributes to the aroma’s formation through the production of important flavor compounds such as diacetyl, other carbonyls, ethyl acetate, and isoalcohols [7,15,16].

Although sourdoughs have been widely studied, currently, there is an interest in exploring their microbial composition, due to the numerous scientific studies that highlight their positive influence on sensory, nutritional, and shelf-life characteristics of naturally leavened products [17,18,19]. Additionally, the continuous search for starter cultures with new attributes, aimed at enhancing the nutritional and nutraceutical traits of sourdoughs, is driving the interest in exploring their microbial diversity [20,21,22].

In the present work, we investigated the yeasts and LAB population of sourdoughs for Panettone production, as well as the volatile fraction of dough samples, with the aim to select candidate strains for developing single and/or multiple starter cultures.

## 2. Materials and Methods

### 2.1. Panettone Production and Sample Collection

Two sourdoughs, defined as MA and MB, were used to produce an artisanal Panettone in a bakery (Panificio Fantuzzi) of the Emilia Romagna region (Italy). They differed in the kind of storage after the periodical refreshments; specifically, MA was kept in a cotton bag, while MB in a bucket with water. Starting from these, the refreshment procedures were made using only wheat flour (type 0) and water. Three back-slopping steps were made for MA and only two for MB. In both cases, about 28% (*w/w*) of the final refreshed sourdough was used for the first dough preparation by adding the following ingredients: flour (4.0 kg), sugar (1.25 kg), yolks (1.0 kg), butter (1.5 kg), and water (2.1 L). After 18 h of incubation at 20–22°C, a second dough was prepared by mixing the first dough with flour (1.5 kg), sugar (1.0 kg), yolks (1,0 kg), butter (2.0 kg), barley malt (0.05 kg), salt (0.065 kg), and water (0.25 L). The final dough was left to rise for 5–6 h before baking. No aromatic ingredients were added.

**Figure 1:**
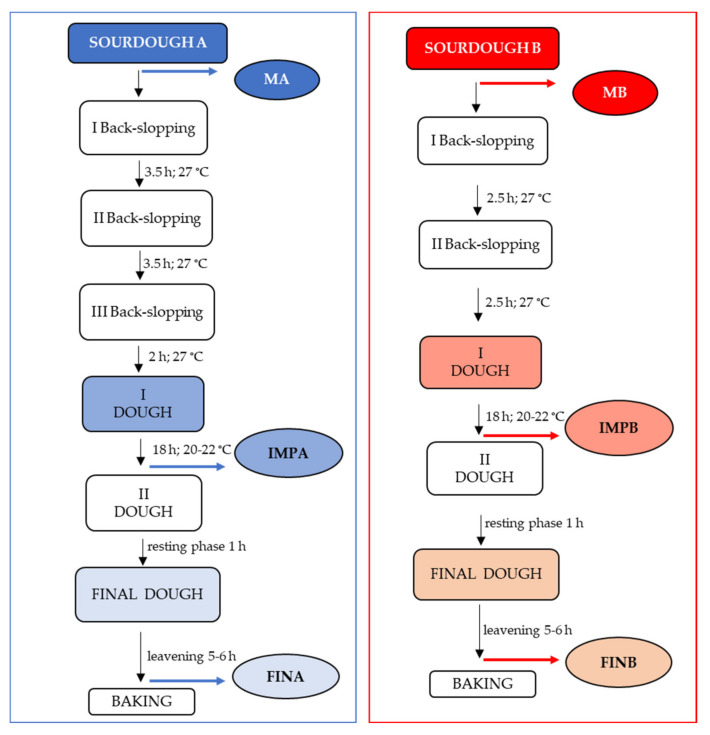
Artisanal Panettone production using two different sourdoughs (MA and MB). The samples highlighted were collected concurrently at the same stages for their investigation.

During the entire process, six samples were taken concurrently in sterile containers for the laboratory analyses. Specifically, the samples were MA and MB; the first doughs were named IMPA and IMPB, collected after the leavening of 18 h, and the final doughs were called FINA and FINB, collected just before the cooking. A schematic representation of the process with the sampling points is reported in Figure 1.

### 2.2. Determination of Physicochemical Parameters

The following parameters pH, total titratable acidity (TTA), and water activity (a_w_) were evaluated on all the samples collected. In detail, pH was detected with a pH meter XS series pH 70 (Bormac srl, Carpi, Italy), and TTA was determined on the homogenized samples and expressed as the amount (mL) of 0.1 N NaOH necessary to achieve pH 8.3 [23]. The a_w_ was measured with the AquaLab 4TE instrument (Meter Group, Pullman, WA, USA) following the manufacturer’s instructions. Each sample was analyzed in triplicate.

### 2.3. Yeasts and LAB Isolation

To obtain microbial isolates, 10 g of each sample was collected and mixed with 90 mL of physiological solution (9 g/L of NaCl) in a Stomacher bag and homogenized for 2 min. From the homogenate samples, serial dilutions from 10^-1^ to 10^-7^ were made and appropriate dilutions were plated in triplicate on different agar media. Specifically, Yeast Peptone Dextrose Agar (YPDA; 10 g/L yeasts extract, 10 g/L peptone, 20 g/L dextrose, and 20 g/L agar) supplemented with 0.1 g/L of chloramphenicol (Sigma-Aldrich, Milan, Italy) was used for yeasts isolation. Plates were incubated at 27 °C for 2 days. LAB isolation was made on de Man Rogosa Sharpe (MRS agar; Oxoid, Milan, Italy) and Sourdough Bacteria Agar (SDB; 6 g/L trypticase, 3 g/L yeast extract, 20 g/L maltose, 3 mL of 10% Tween 80, 1.5 % of fresh yeast extract, 15 g/L agar), both supplemented with 0.1 g/L cycloheximide (Sigma-Aldrich). Plates were incubated in jars with the Anaerogen system (Oxoid) at 30 °C for 3 days. After the colonies were counted on the plates of each medium, between 3 and 10 colonies were randomly selected at the highest dilutions and purified by streaking on the correspondent isolation medium. Basic phenotypic tests, such as Gram staining and catalases, were done for bacteria. Cell morphology of the isolates was observed using a Zeiss Axiolab microscope (Carl Zeiss Ltd., Cambridge, UK). After the molecular characterizations, yeasts and bacteria strains were preserved in the Unimore Microbial Culture Collection (UMCC) in accordance with standard procedures [24]. A seed lot of the strains was stored at −80 °C, in cryovials containing an aliquot of each culture, grown in liquid medium, and 25% (*v/v*) glycerol (Carlo Erba, Milan, Italy).

### 2.4. DNA Extraction and Molecular Characterization

Genomic DNA from yeast and bacterial cultures was extracted following the protocols of Hoffman and Winston [25] and Ausubel et al. [26], respectively. The quality and concentration of the extracted DNA were checked by NanoDrop™ 1000 Spectrophotometer (Thermo Fisher Scientific, Waltham, MA, USA).

For a preliminary characterization at species level, PCR-RFLP of the internal transcribed spacer (ITS) region and 5.8S gene of rDNA was performed on yeast gDNA following the protocol described by Pulvirenti et al. [14]. ITS1 (5′-TCCGTAGGTGAACCTGCGG-3′), ITS4 (5′-TCCTCCGCTTATTGATATGC-3′) oligonucleotide primers (Eurofins MWG Operon, Ebersberg, Germany), and PCR amplification kit, which included TaKaRa Taq DNA Polymerase (Takara Bio, Inc., Otsu, Shiga, Japan), were used. PCR was made on a final volume of 50 µL by using the thermal cycler BioRAD T100^TM^ (Bio-Rad Laboratories, Milan, Italy). Amplicons digestion was performed with *Hae*III restriction endonucleases (Fermentas, Hanover, ND, USA), according to supplier’s recommendation. The fragments were separated on 2% (*w/v*) agarose gel electrophoresis in 0.5X Tris-Borate-EDTA (TBE) buffer at 90 volts for 2 h. The gels were stained with SYBR Safe reagent (Thermo Fisher Scientific) and visualized on the Transilluminator Safe View (Cleaver Scientific LTD, Rugby, UK).

A first rapid detection of LAB strains belonging to *Fl. sanfranciscensis* species was made based on PCR amplification of the 16S–23S rDNA intergenic spacer region (ISR). PCR was performed according to the protocol described by Valcheva et al. [27] by using the oligonucleotide primers 16S p2: (5′-CTTGTACACACCGCCCGTC-3′) and 23S p7: (5′-GGTACTTAGATGTTTCAGTTC-3′) (Eurofins MWG Operon). The fragments were separated on 1.5% (*w/v*) agarose gel electrophoresis in 0.5X Tris-Borate-EDTA (TBE) buffer at 90 volts for 1.5 h.

For strain characterization, repetitive element sequence-based (rep)-PCR genotyping [28] using (GTG)_5_ oligonucleotide primer (Eurofins MWG Operon) was performed on gDNA of all the isolates according to the protocol described by La China et al. [29]. Fingerprinting profiles were assessed after electrophoresis on 1.5% (*w/v*) agarose gel, carried out as previously described. Pattern band lengths were determined by comparison against a 100 bp plus DNA ladder (Thermo Fisher Scientific).

Digitalized images of the electrophoretic profiles were analyzed by using the GelCompare software v3.0 (Applied Maths, Kortrijk, Belgium). The similarity matrix of the bands’ patterns was computed by using Pearson’s correlation with optimization and curve smoothening values at 0.5%. Dendrograms were constructed by the unweighted-pair group method using arithmetic averages (UPGMA) clustering method.

### 2.5. Species Assignment and Phylogenetic Clustering

At least one strain of yeasts and bacteria isolated from each sample was subjected to sequencing analysis for the species assignment and the successive phylogenetic clustering. To this aim, targeted regions were amplified using specific primer sets. In particular, the D1/D2 region of 26 rRNA large subunit from the yeast gDNA was amplified using primers NL1 (5′-GCATATCAATAAGCGGAGGAAAAG-3′) and NL4 (5′-GGTCCGTGTTTCAAGACGG-3′), as reported by Kurtzman et al. [30].

Yeast Ab1 files obtained from the sequencing were processed using CodonCode aligner to trim the sequences based on phred score (phred 20). The sequence ends were trimmed according to primer length. High-quality sequences were aligned against YeastIP database [31]. Reference sequences were downloaded from YeastIP database, based on matches, e-value, and identity scores.

Regarding bacteria, amplification of 16S rRNA gene was performed using primers 27f (5′-CTGGGATCCATTTACTCGAGAGTTTGATCCTGGCTCAG-3′) and 1490r (5′-GGTTCCCCTAAGCTTACCTTGTTACGACTTC-3′), as reported by Sato et al. [32].

PCR amplicons were purified and cleaned using ExoSAP PCR cleanup reagent (Thermo Fisher Scientific) and sequenced by Eurofins MWG Biotech Company (Ebersberg, Germany). The strains sequenced were deposited in the UMCC Database (https://umcc.bio-aware.com/, accessed on 20 April 2021).

Bacteria Ab1 files were processed as described for the yeast dataset. High-quality sequences were aligned against 16S rRNA sequence database from NCBI using blast algorithm. The top hit with the highest percentage of identities (considering a minimum threshold of 97%) was downloaded and used as reference.

The sequences of selected strains and references were aligned all-vs.-all using Clustal W v2.1 [33]. For constructing the phylogenetic trees, the multiple sequences alignment was imported into MegaX v10.2 and trimmed to match the sequence length. The phylogeny was inferred computing a neighbor-joining phylogenetic tree based on 1000 bootstraps for both yeasts and LAB dataset. The Tamura-Nei DNA evolutionary model was used, applying a discrete Gamma distribution to model evolutionary rate differences among sites [34].

Newick trees of both bacteria and yeasts were visualized using Interactive Tree of Life (ITOL) v4 [35] and rooted at outgroup reference strains. The sequences of D1/D2 region and 16S rRNA gene were submitted to GenBank/EMBL/DDBJ under the accession numbers from MZ170795 to MZ170810 and from MZ170701 to MZ170718, respectively.

### 2.6. HS-SPME-GC/MS Profiling of Fermented Products

Volatile organic compounds (VOCs) developed during dough fermentation were determined by headspace solid-phase microextraction (HS-SPME) followed by gas-chromatography/mass spectrometry (GC-MS) analysis.

Seven grams of the six samples collected were weighted into 25 mL screw-cap glass vials provided with Mininert© valves.

Vials were conditioned at 60 °C for 30 min in a thermoblock (Falc Instruments, Treviglio, Italy), and then a divinylbenzene/carboxen/polydimethylsiloxane (DVB/CAR/PDMS) SPME fiber was exposed in the headspace for 30 min at the same temperature for the extraction of volatile compounds. Chromatographic separation of analytes was carried out by an Agilent (Paolo Alto, CA, USA) 6890N GC followed by mass spectrometer Agilent 5973 Network Mass Selective Detector. After extraction, fibers were desorbed for 3 min into the GC injector port set in splitless mode at 240 °C. The GC carrier gas used was helium at 1 mL/min and the detector temperature set at 240 °C. GC oven temperature program was: start at 50 °C for 3 min, 5 °C/min until 160 °C, hold at 160 °C for 2 min, 20 °C/min until 240 °C, hold at 240 °C for 2 min. Peaks identification was carried out by comparison with system libraries (Wiley, Nist). The analyses were performed in triplicate. A correlation analysis of VOCs and species detected in sourdough and dough samples was obtained using the Corrplot v. 0.84 [36] package implemented in R v 4.0.3 [37].

### 2.7. Statistical Analysis

pH, temperature, TTA, and a_w_ data were expressed as mean value ± standard deviation (sd). Microbial counts were converted to log colony-forming units (CFU) per g of sample and expressed as mean ± sd. The data were subjected to one-way analysis of variance (ANOVA) followed by Tukey HSD post hoc test to establish significant differences between means (*p* < 0.05; n = 3). For the statistical analysis, the software GraphPad Prism version 8 was used (GraphPad Software, San Diego, CA, USA).

Total VOCs in sourdough and dough samples were expressed as a mean value of the total chromatographic area detected by GC-MS analysis, while the relative abundance of each VOC was reported as % of the total chromatographic area ± sd.

## 3. Results

### 3.1. Samples Analysis and Microbial Counts

The physicochemical parameters detected on the samples collected during the Panettone production are reported in Table 1. The pH values of sourdoughs MA and MB pH were 3.90 and 3.81, respectively. A significant increase in the pH was observed in all the other samples because of the dilution effect of the ingredients added during the production process. Accordingly, the TTA was higher in the two sourdoughs and decreased in doughs IMPA and IMPB, as well as in the final doughs. The same trend was observed also for the water activity (a_w_), which reached a final value of 0.9441 and 0.9411 in FINA and FINB, respectively. In these cases, above the dilution effect, the addition of ingredients, such as sugars, also contributed to lowering the a_w_ values [8].

Regarding the colony counts of the yeasts assessed on YPDA, it ranged from 6.81 to 7.83 log cfu/g for samples A and from 7.56 to 7.89 log cfu/g for B. The viable bacterial population enumerated on MRS and SDB media was generally higher in MA and MB, with values around 6 log cfu/g; in all the other samples, the values were around 5 log cfu/g (Table 2). In total, 57 yeast isolates were collected and further investigated. Concerning the isolates from MRS and SDB media, 20 were selected as presumptive LAB after the basic microbiological screenings.

### 3.2. Yeasts’ Molecular Characterization and Phylogenetic Analysis

The analysis of the RFLP-PCR of ITS-5.8S rDNA and the yeasts’ amplicons digestion with *Hae*III allowed us to detect two different profiles (Table 3). Specifically, all the isolates belonging to samples A showed the same profile characterized by two bands of 230 and 400 base pairs (bp). As reported in the literature, this profile was tentatively attributed to the species *K. humilis* (formerly *Candida milleri*) [13,14]. Regarding the isolates from samples B, 12 showed a profile with two bands, as before, whereas a profile with three bands of 150, 180, 230, 320 bp were detected in the remaining ones. These isolates were preliminarily attributed to *S. cerevisiae* species according to Esteve-Zarzoso et al. [38].

**Figure 2:**
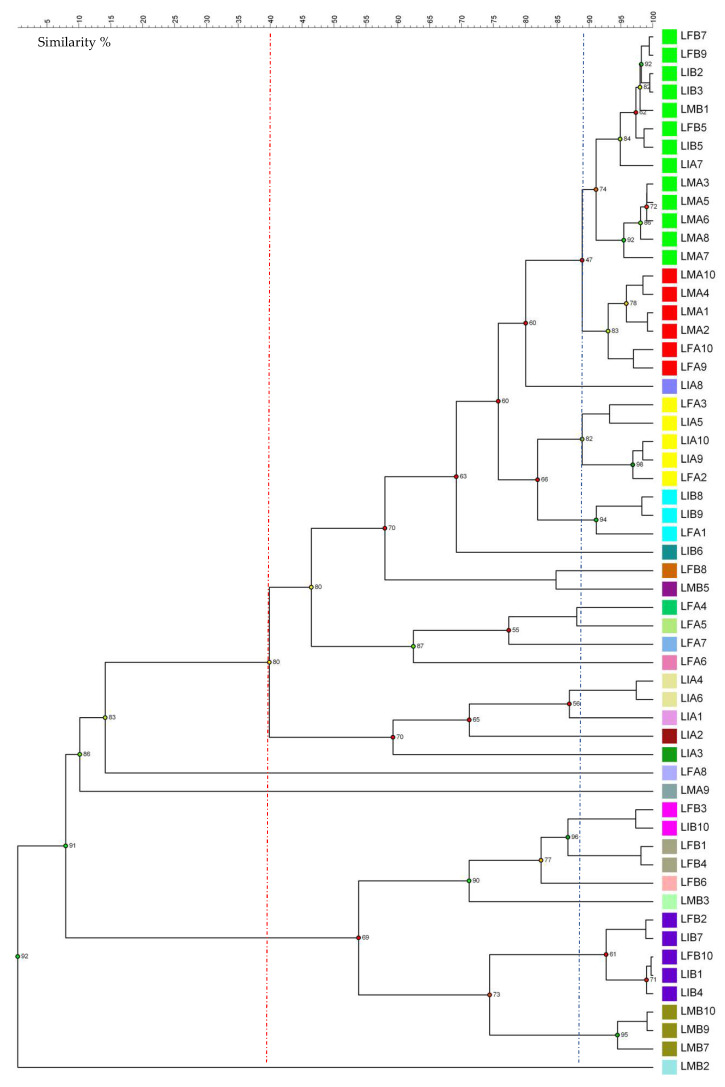
Dendrogram obtained from unweighted pair group method with arithmetic mean (UPGMA) analysis, using Pearson’s coefficient, of the (GTG)_5_ rep-PCR patterns. The similarity threshold for biotypes discrimination was 89% (blue line). The cophenetic coefficient is represented by numbers and dots colored as red, orange, yellow, or green, based on the branch quality. Two major clades are distinct with a threshold of 40% (red line).

For strain clusterization, the (GTG)_5_ rep-PCR fingerprinting techniques were performed. Figure 2 reports the dendrogram obtained from UPGMA analysis using Pearson’s coefficient. The discrimination of biotypes was performed considering a similarity threshold of 89%, which allowed the grouping of yeast isolates into 25 clades. The resulting discrimination power, calculated using the Simpson’s index, was 0.92. The clades with the highest number of strains (from 5 to 13) included those preliminarily attributed to *K. humilis*. Considering a similarity threshold of 40%, all the tentative *K. humilis* strains (42 in total) were grouped closely in a major clade clearly distinct from a minor clade that included the remaining 15 strains preliminarily attributed to *S. cerevisiae.*

**Figure 3:**
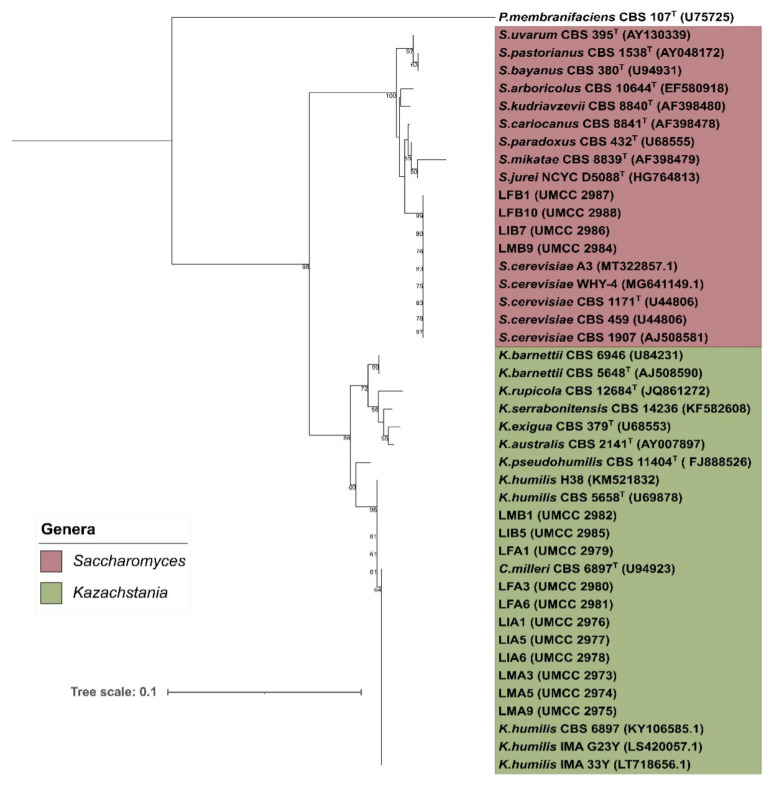
Neighbor-joining phylogenetic tree representing the phylogenetic distances of isolates from sourdough MA and MB and the strain types of identified yeasts species. The tree was made using Tamura-Nei model, and a discrete Gamma distribution was used to model evolutionary rate differences among sites. Node numbers indicate bootstrap values obtained using 1000 replicates.

A total of 43 high-quality sequences were used to infer the phylogeny of yeast isolates. Reference sequences belong to *Saccharomyces* and *Kazachstania* genera, detected after alignment in the YeastIP database. After all-vs.-all sequence alignment, sequences were trimmed all at the same length, resulting in a total length of 599 bp. The neighbor-joining phylogenetic tree was reported in Figure 3. The bootstrap values higher than 50%, used to define the accuracy of branch prediction, were reported.

According to sequence clusterization, the phylogenetic tree resulted in three well-defined clades, distinguishing with high accuracy the detected species, as reported by bootstrap values. The total average of dissimilarities among all phylotypes considered was 7.04%. The two major clades included *Saccharomyces* and *Kazachstania* species, supported by a bootstrap value of 98%. A total of four isolates, coming from sample B (LFB1, LFB10, LIB7, and LMB9), were grouped in *S. cerevisiae* clade, having a percentage of identity of 100% with *S. cerevisiae* CBS 1171^T^. The remaining *Saccharomyces* species were grouped in three different clades, of which one includes *S. uvarum* CBS 395^T^ and the hybrid species from *S. uvarum*, *S. bayanus* CBS 380^T,^ and *S. pastorianus* CBS 1538^T^. Another cluster of *Saccharomyces* species includes *S. kudriavzevii* CBS 8840^T^ and *S. arboricolus* CBS 10644^T^, which share 99.2% of sequence identitt. The remaining clade of *Saccharomyces* includes *S. cariocanus* CBS 8841^T^, *S. paradoxus* CBS 432^T^, *S. jurei* NCYC D5088^T^, and *S. mikatae* CBS 8839^T^. Species included in this clade were phylogenetically closest, except for *S. mikatae*, sharing a dissimilarity percentage of about 1% with the other clade members.

The *Kazachstania* clade is branched out into three subclades. A clade including isolates coming from sample A and sample B was represented by *K. humilis*. Three isolates, represented by LMB1, LIB5, and LFA1, share 100% identity with *K. humilis* CBS 5658^T^ and *K. humilis* H38. The remaining strains in the *K. humilis* clade, including *C. milleri* CBS 6897 (currently a synonym of *K. humilis*), are phylogenetically quite distant from *K. humilis* CBS 5658^T^, which shares the 99.8% similarity, indicating a low divergence as also reported from different studies [39,40]. Given this consideration, *K. humilis* and *C. milleri* are considered conspecific [41]. The most closely related to *K. humilis* strains is *K. pseudohumilis* CBS 11404^T^, with divergence supported by a bootstrap of 60%. The remaining *Kazachstania* strains included in the dataset were in a different clade, supported by a bootstrap of 86%. This clade includes *K. barnettii* CBS 5648 and *K. barnettii* CBS 6946^T^, forming a single group and the stains *K. rupicola* CBS 12684^T^, *K. serrabonitensis* CBS 14236^T^, *K. australis* CBS 2141^T^, and *K. exigua* CBS 379^T^. The represented species are low in divergence since the sequence differs by 0.7% to 1.7%.

### 3.3. Bacteria Molecular Characterization and Phylogenetic Analysis

The analysis of the 16S-23S rDNA ISR allowed to detect *Fl. sanfranciscensis* species among the bacterial isolates (Table 4). In particular, PCR products from all the MA and MB isolates yielded similar profiles containing three bands of 600, 700, and 800 bp; these isolates were preliminarily attributed to *Fl. sanfranciscensis* [27]. All the remaining isolates showed a profile with only two bands of 600 and 800 bp. This result was consistent with previously reported data for *Lactobacillus* 16S–23S ISR organization [27,42,43].

**Figure 4:**
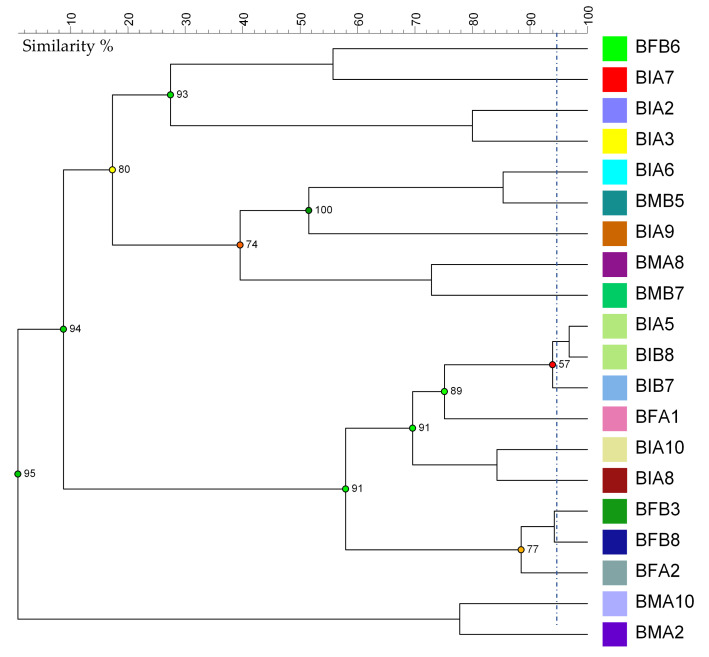
Dendrograms obtained from unweighted pair group method with arithmetic mean (UPGMA) analysis, using Pearson’s coefficient, of (GTG)_5_ rep-PCR. The similarity threshold for biotypes discrimination was 95% (blue line). The cophenetic coefficient is represented by numbers and dots colored as red, orange, yellow, or green, based on the branch quality.

The digitized patterns obtained from (GTG)_5_ rep-PCR, analyzed with the UPGMA method and using Pearson’s coefficient, allowed the construction of the dendrogram shown in Figure 4. The biotypes discrimination was performed considering a similarity threshold of 95%, which allowed the grouping of the strains into 19 different clades; only the strains BIA5 and BIB8 were included in the same clade. The calculated Simpson’s index was 0.99, indicating a high diversity among the isolated strains including those preliminarily attributed to the species *Fl. sanfranciscensis*. This is consistent with the evidence reported by several authors that highlighted the great variability among *F. sanfranciscensis* strains [7,27].

High-quality sequences obtained from the bacteria dataset were analyzed in order to determine the phylotypes of isolates. After all-vs.-all alignment, sequences were trimmed at the same length, resulting in a total length of 904 bp. Reference sequences were downloaded from NCBI 16S rRNA database, resulting in two genera including *Leuconostoc* and *Lactobacillus*. The sequence of *Bacillus albidus* MCCC 1A02146^T^ was used as an outgroup.

**Figure 5:**
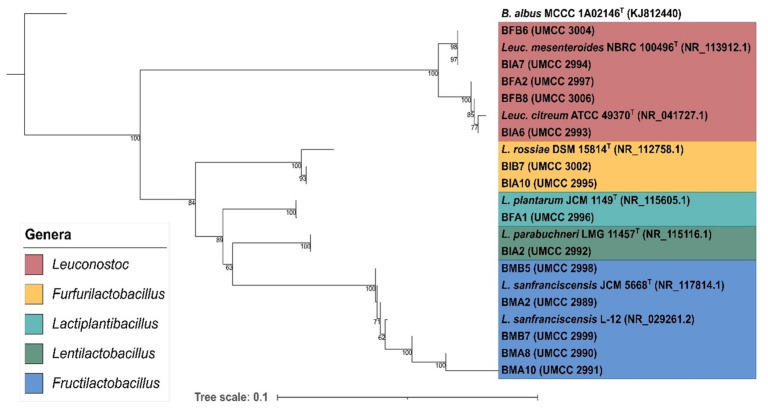
Neighbor-joining phylogenetic tree obtained by 16S multiple alignment. A gamma distribution was applied to model the rate variation among sites. The node numbers indicate the percentage of replicates in which taxa clustered at the same distance (bootstrap test). References species used in the dataset are reported, in the figure, using their former name and the associated NCBI id. Given the recent re-organization of the Lactobacillus genus, the different genera are highlighted with different colors and named using the new taxonomical nomenclature, according to Zheng et al. [11].

The average phylogenetic distance among all sequences included in the dataset was 13.17%. The resulting phylogenetic tree was represented in Figure 5. A total of two major clades are depicted, represented by the clade of *Leuconostoc* genus and the clade including the genera *Furfurilactobacillus*, *Lactiplantibacillus*, *Lentilactobacillus,* and *Fructilactobacillus*. In particular, *Leuconostoc* clade includes five isolates and is branched in two subclades, *Leuc. mesenteroides* NBRC 100469^T^ and *Leuc. citreum* ATCC 49370^T^.

Two strains, BIA7 and BFB6, were grouped with *Leuc. mesenteroides* NBRC 100469^T^, sharing 100% similarity.

Three strains, BFA2, BFB8, and BIA6, were clustered with *Leuc. citreum* ATCC 49370^T^, sharing 100% of similarity among them and 99.9% of similarity with the reference strain. The phylotype classification was supported by high bootstrap values (ranging between 77% and 100%).

The remaining isolated strains were included in the other major clade, represented by four species: *Furfurilactobacillus rossiae* (formerly *Lactobacillus rossiae*) DSM 15814^T^, *Lacp. plantarum* JCM 1149^T^, *Lentilactobacillus parabuchneri* (formerly *Lactobacillus parabuchneri*) LMG 11457^T^, and *Fl. sanfranciscensis* JCM 5669^T^. Branches in this clade, representing species differentiation, were supported by high bootstrap values, ranging between 63% and 100%.

Specifically, BIA10 and BIB7 strains were grouped with *Furl. rossiae* DSM 15814^T^. The two strains shared 100% of identity and were phylogenetic divergent with the reference strain of 1.51%. The strain BFA1 was identified as *Lacp. Plantarum,* having 100% sequence similarity to *Lacp. plantarum* JCM 1149^T^. The branching was supported by 100% of bootstrap value, meaning an inference with high confidence. Isolate BIA2 clustered with *Lenl. parabuchneri* LMG 11457^T^, supported by a 100% bootstrap value and sharing 100% identity. The remaining strains, represented by BMB5, BMA2, BMB7, BMA8, and BMA10, were included in the *Fl. sanfranciscensis* clade (bootstrap value 100%). All references and isolates showed low divergence, since the maximum phylogenetic distances observed was 0.2% in the case of BMB10 with BMA8. Given the high sequence similarities and the high confidence represented by the bootstrap values, it is reasonable to assume that all of the isolates belonging to *Fl. sanfranciscensis* clade could be assigned to this species. Our results are consistent with previous evidence that reported the remarkable polymorphism of the 16S rDNA within the *Fl. sanfranciscensis* species and placed this microorganism in the *Lacp. plantarum* group based on the 16S rDNA phylogenetic analysis [27].

### 3.4. Volatile Organic Compounds Profiling

The VOCs profiling of sourdoughs and following dough samples revealed significant quantitative and qualitative differences, which can be attributed to the different microflora of each of the two systems A and B, given that the ingredients used were the same.

**Figure 6:**
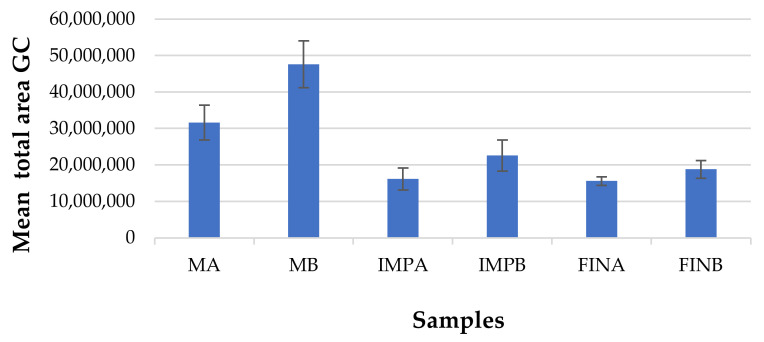
Mean total chromatographic area of the identified volatile organic compounds (VOCs) in sourdoughs and dough samples A and B.

Sourdoughs were characterized by much higher total VOCs, as can be inferred from Figure 6. Indeed, the volatiles produced in the sourdough undergo a dramatic dilution from the addition of ingredients. It is noteworthy that the significantly higher (1.5-fold) VOCs levels, expressed as total chromatographic area, observed in MB (4.76 × 10^7^) compared with MA (3.16 × 10^7^), are followed by significantly higher levels also recovered in IMPB (2.26 × 10^7^) and FINB (1.88x10^7^) samples compared with IMPA (1.62 × 10^7^) and FINA (1.56 × 10^7^), respectively, even if the difference is slightly reduced to 1.2-fold.

**Figure 7:**
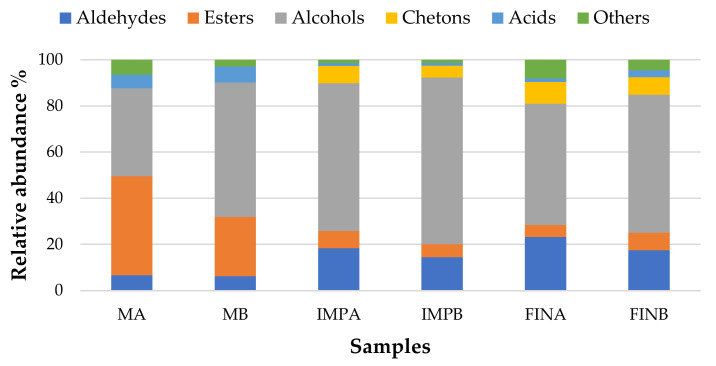
Relative abundance (%) of the chemical classes of volatile organic compounds (VOCs), determined in sourdough and dough samples at different steps of *Panettone* preparation.

The relative abundance of chemical classes of VOCs in the volatile fraction of samples is shown in Figure 7. As much as >80% of the volatile fraction of sourdoughs was made up of alcohols and esters together, while aldehydes and acids each represented about 6-7% of the total chromatographic area.

Interestingly, the VOCs profile of MA was characterized by a balanced level of esters and alcohols, while in MB samples the alcohols prevailed; this finding agrees with the presence of *S. cerevisiae* in B samples. The relative composition of the volatile fraction changed after formulation and leavening, the most noteworthy changes being the increase in aldehydes from 6–7% to 17–23% of the total VOCs, a reduction in esters to 5–7%, and the development of ketones, represented by acetoin (3-hydroxy, 2-butanone), which was not detected in the sourdough samples.

The main aliphatic aldehydes found were acetaldehyde, characterizing the VOC profile of all samples and showing an evident increasing trend from MB to FINB samples (Table 5). To a lower extent, there were 2-butenal, only found in fermented dough samples, hexanal, the product of lipid oxidation by the lipoxygenase pathway [44], and heptenal, retrieved only in MA and MB samples; they accounted for about 4% and 0.3–0.7%, respectively. These aldehydes could not be detected in further steps, according to previous reports [16]; however, the hexanal loss was not followed by a corresponding 1-hexanol increase during maturation.

Among aromatic aldehydes, phenylacetaldehyde accounted for about 5.5–7.6% of total VOCs in dough samples, while benzaldehyde accounted for 0.5–2%. The B samples always showed higher relative abundances of these compounds, while none of them were detectable in sourdough samples, suggesting the contribution of dough ingredients in their formation.

Among esters, ethyl acetate represented about 40% and 21% of the total VOCs of sourdoughs MA and MB, respectively, followed by isoamyl acetate (about 2%), ethyl lactate (1.5–2%), and ethyl hexanoate, while their concentration was dramatically reduced in the leavened products. This finding agrees with previous data on the natural fermentation of bakery products [16], which report ethyl acetate relative abundances ranging from 16 to 27% in sourdough, falling to about 3% in leavened products.

Ethyl butanoate, which was not detected in sourdoughs, was determined in both leavened products, at about 0.8-0.9% of total VOCs.

VOCs belonging to the alcohols group were dominant, with ethanol being the most concentrated, followed by isoamyl alcohol and phenethyl alcohol. Isoamyl alcohol, ranging from 6.4 to 8.9% of total VOCs in the dough samples, was also present at comparable levels in the sourdough samples and always at higher relative abundance in B samples: this alcohol is considered the most impacting aroma component produced by yeasts, together with isobutanol [45]. Phenethyl alcohol represented only 0.5% of VOCs in MA, while it was more concentrated in MB (3.3%); the relative abundance of this alcohol increased with the production steps, representing 3.9 and 8.2% of total VOCs in FINA and FINB, respectively. As for ethanol, phenethyl alcohol was always more concentrated in B samples. Isobutanol was not detected in sourdough samples but represented 2.5% of total VOCs in FINB, while it was not retrieved in FINA.

Interestingly, MA and MB samples did not show ketones among VOCs, while IMPA and IMPB, as well as FINA and FINB, revealed the presence of acetoin (3-hydroxy-2-butanone) up to about 8% of total VOCs: this compound can be attributed to both fermentation by *S. cerevisiae* and homofermentative LAB [46] and to the contribution of butter in the dough formulation. According to Montanari et al. [16], acetoin was not retrieved in sourdough, but could only be detected in the leavened dough at higher levels in dough samples A.

### 3.5. Correlation of VOCs and Species Detected in Sourdough and Dough Samples

**Figure 8:**
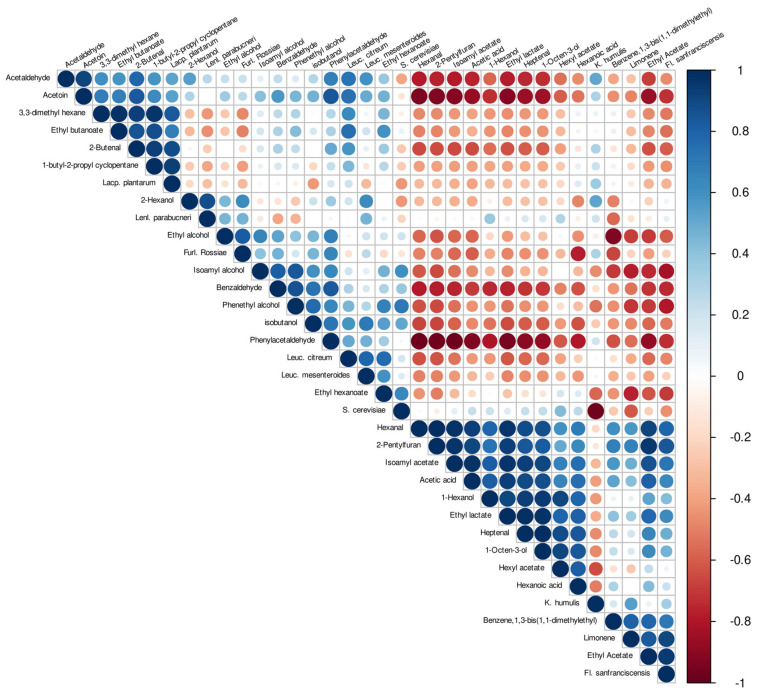
Correlation plot among discovered VOCs and isolated species, obtained using R package Corrplot and Pearson’s correlation index. The positive and negative correlation were considered based on R^2^ ≥ 0.6 and R^2^ ≤ −0.6, respectively.

To understand the correlation among the detected VOCs and bacteria or yeasts isolated a Pearson’s correlation index was calculated. The correlation plot is reported in Figure 8. Considering bacteria isolates, the major contributor to the aroma composition was attributed to *Fl. sanfranciscensis*, which was shown to be positively correlated (R^2^ ≥ 0.6; *p* < 0.05) with a wide range of VOCs belonging to four classes, such as aldehydes, esters, alcohols, and acids, as well as with a minor compound as limonene. Two other species, specifically *Leuc. citreum* and *Lacp. plantarum*, contributed in a moderate way to the aroma production, resulting in each of them being positively correlated to four different compounds. In particular, *Leuc. citreum* was correlated to acetaldehyde, ethyl butanoate, ethyl hexanoate, and acetoin (R^2^ ≥ 0.6; *p* < 0.05). In the case of *Lacp. plantarum*, the major contribution was related to 3,3-dimethyl hexane, ethyl butanoate, 2-butenal, and 1-butyl-2-propyl cyclopentane. Interestingly, *Furl. rossiae* was shown to have a good correlation to ethyl alcohol production (R^2^ = 0.83; *p* < 0.05). The other two species detected, *Lenl. parabuchneri* and *Leuc. mesenteroides*, were significantly correlated with just one compound, precisely 2-hexanol (R^2^ = 0.87; *p* < 0.05) and isobutanol (R ^2^ = 0.71; *p* < 0.05), respectively. Regarding the yeasts detected, *K. humilis* and *S. cerevisiae*, their contribution in terms of VOCs was shown to be, generally, very low. In particular, *S. cerevisiae* was found to be positively correlated with isoamyl alcohol, ethyl hexanoate, phenethyl alcohol. No relevant correlation was highlighted for *K. humilis*.

## 4. Discussion

In the present work, the viable microbial population of different dough samples collected during the production process of Panettone was assessed.

The sourdough MA showed a strong dominance of *K. humilis* species, with only one sample identified as a group-species *K. humilis/K. parahumilis.* This homogeneous result was already observed in traditional sourdoughs [8,10,13,47]. In sourdough MB, four sequenced strains belonged to the *S. cerevisiae* species, while the other strains belonged to the *K. humilis* species. The interactions of these two yeast species were also usually found in several traditional sourdoughs [8,48,49]. In our case, the minor number of refreshments of MB sample and the reduced fermentation step probably could have favored the dominance of *S. cerevisiae*, thanks to its rapid metabolism and growth rate.

Concerning LAB, the results showed the prevalence of heterofermentative-obliged bacteria, with the exception of *Lacp. plantarum*, which is a facultative heterofermentative bacterium, normally colonizing sourdoughs characterized by continuous back-slopping and low incubation temperature [3]. *Fl. sanfranciscensis* was found in both sourdoughs with a major occurrence in the A samples. Strains of this species are commonly reported as the main components of sourdoughs due to their ability to adapt to the highly selective conditions of this environment [4,6,18,49]. Moreover, *Fl. sanfranciscensis* strains utilize preferentially maltose and are generally unable to ferment fructose; therefore, there is a non-competitive association of this species with other different LABs. For instance, a stable association is reported between *Fl. sanfranciscensis* and *Lacp. Plantarum,* as the latter species preferentially ferments glucose and fructose rather than maltose, whose metabolism is subject to carbon catabolite repression [3,50,51]. Regarding maltose utilization, another stable association is described between *Fl. sanfranciscensis* and maltose-negative yeasts such as *K. humilis*. On the other hand, the interactions of *Fl. sanfranciscensis* and *S. cerevisiae* are reported to be strain-specific and evidently competitive [52].

Regarding *Furl. Rossiae*, its presence in both sourdough samples is quite interesting. This species was described by Corsetti et al. [53] and subsequently studied for its fermentative properties by Di Cagno et al. [54]. *Furl. Rossiae* was found in both samples A and B, mostly in association with other LAB species, as shown in IMPA. The species *Leuc. Citreum* and *Leuc. Mesenteroides*, which were also found in both dough types, are typical of the last stages of the Panettone process [18]. Their presence, along with *Lacp. Plantarum*, *Furl. Rossiae*, and *Fl. Sanfranciscensis*, could be explained by the low temperature of the process [3]. The occurrence of *Lacp. plantarum* in sourdough is also widely described as a both sub-dominant and dominant species [18,55]. Less common but explainable is the presence of *Lenl. parabuchneri*, a species commonly found in beer and dairy products [55].

Concerning the VOCs detected and their correlation to the yeasts and LAB species found in the samples, the contribution of the microbial activity to the production of the different metabolites was evident.

Noteworthy are the VOCs belonging to the alcohols group. Ethanol, isoamyl alcohol, and phenethyl alcohol were much higher in MB compared to MA and, even though their amount in IMP and FIN samples decreased because of a dilution effect (due to addition of ingredients), they kept higher concentrations in the different stages of method B, with a final difference by around 37% between the two methods. This result may be ascribed to the presence of *S. cerevisiae* in the B samples. Hansen and Schieberle [56] reported that the content of some volatile compounds in baked leavened products such as bread is related to the concentration in the corresponding sourdough, where they are usually more concentrated, since dough recipes imply the use of a fraction of sourdough added to other ingredients. Ethanol represented about 31% and 44% in MA and MB, respectively, thus confirming itself as the principal alcohol in the volatile fraction. Similarly, Montanari et al. [16] reported a relative composition ranging from 37 to 42% in the volatile fraction of sourdough samples, which were of the same origin but obtained with different maturation conditions.

In the present study, the different origins and compositions of the two sourdoughs gave rise to higher differences and to a wider range of variability. In particular, the comparison between the two methods for sourdough preparation highlighted higher total esters content in MA samples; however, isoamyl acetate and ethyl lactate were significantly higher in MB samples (Table 5). Ethyl acetate was detected, though to a lower extent, in the leavened products, decreasing from IMP to FIN samples, while ethyl lactate and isoamyl acetate were not detected; this trend also agreed with the literature [16]. The decrease in ethyl acetate through the production steps was limited in method B, which revealed the occurrence of *S. cerevisiae*.

According to De Luca et al. [57], alcohols, acids, and aldehydes represent the most characteristic VOC families influencing the fruity, green, floral, sweet, alcoholic, and fatty acid odors in bread. The LAB species detected in our samples were differently correlated to the specific VOCs, showing a complementary behavior in some cases. This supports the use of a defined combination of selected strains to achieve the desired aroma production.

## 5. Conclusions

In the present work, the molecular characterization of the dough’s population revealed the presence of the dominant yeasts, *K. humilis* and *S. cerevisiae*, as well as the LAB species, namely *Fl. sanfranciscensis*, *Lacp. plantarum, Furl. rossiae*, *Lenl. parabuchneri*, *Leuc. mesenteroides*, and *Leuc. citreum*.

Results highlight the relevance of refreshment procedures as the main factor in maintaining a stable and active microflora in industrial conditions.

Moreover, the correlation among the selected strains and the VOCs produced in the different samples allowed us to estimate the species contribution to aroma formation.

Therefore, the research of sourdoughs’ selected strains, with known metabolic properties and high technological performances, is fundamental for their exploitation as starter cultures capable of controlling the manufacturing of sourdoughs. A pool of LAB and yeasts of this study deposited at the UMCC culture collection comprises candidate strains for the development of single and multiple selected starters for Panettone production.

## Figures and Tables

**Table 1:** Physicochemical parameters detected on the samples collected during the Panettone production.

Samples	pH	Water Activity (a_w_)	Total Titratable Acidity (mL NaOH 0.1N/10 g)
MA	3.90 ± 0.03 ^d^	0.0.9855 ± 0.002 ^d^	10.85 ± 0.45 ^b^
IMPA	5.44 ± 0.05 ^b^	0.0.9636 ± 0.001 ^b^	3.35 ± 0.25 ^a^
FINA	5.35 ± 0.02 ^bc^	0.0.9441 ± 0.002 ^a^	3.55 ± 0.45 ^a^
MB	3.81 ± 0.02 ^d^	0.0.9894 ± 0.001 ^d^	10.45 ± 0.45 ^b^
IMPB	5.30 ± 0.07 ^ac^	0.0.9562 ± 0.002 ^c^	3.45 ± 0.30 ^a^
FINB	5.21 ± 0.03 ^a^	0.0.9411 ± 0.003 ^a^	4.05 ± 0.05 ^b^

Values are means ± standard deviation of three replicates (n = 3). Values within a column with different superscript letters are significantly different (*p* < 0.05).

**Table 2:** Colony counts on the growth media used for the isolation of yeasts and lactic acid bacteria from dough samples.

Samples	Means Counts * on the Isolation Media		
	YPDA	MRS	SDB
MA	6.81 ± 0.25 ^b^	6.15 ± 0.14 ^ab^	6.17 ± 0.21 ^b^
IMPA	7.81 ± 0.05 ^a^	5.39 ± 0.12 ^a^	5.65 ± 0.21 ^ab^
FINA	7.83 ± 0.17 ^a^	5.46 ± 0.07 ^a^	5.45 ± 0.19 ^ab^
MB	7.56 ± 0.03 ^a^	6.87 ± 0.42 ^b^	6.16 ± 0.15 ^b^
IMPB	8.02 ± 0.01 ^a^	5.57 ± 0.06 ^a^	5.27 ± 0.09 ^a^
FINB	7.89 ± 0.17 ^a^	5.64 ± 0.04 ^a^	5.64 ± 0.02 ^ab^

* expressed as log of colony forming unit (cfu)/ g of sample ± standard deviation. Values are means ± standard deviation of three replicates (n = 3). Values within a column with different superscript letters are significantly different (*p* < 0.05). YPDA medium (yeast extract, peptone, dextrose, agar); MRS medium (de Man Rogosa Sharpe); SDB medium (Sourdough Bacteria agar).

**Table 3:** RFLP-PCR of ITS-5.8S rDNA.

Sample	Strains *	Size of Amplicon (bp)	Amplicons Digestion with *Hae*III (Size Fragments, bp)
MA	LMA2, **LMA3 (UMCC 2973)**, LMA1, LMA4, **LMA5 (UMCC 2974)**, LMA6, LMA7, LMA8, **LMA9 (UMCC 2975)**, LMA10	630	400–230
IMPA	**LIA1 (UMCC 2976)**, LIA9, LIA10, LIA2, LIA3, LIA4, **LIA5 (UMCC 2977)**, LIA7, **LIA6 (UMCC 2978)**, LIA8	630	400–230
FINA	**LFA1 (UMCC 2979)**, LFA2, **LFA3 (UMCC 2980)**, LFA4, LFA5, **LFA6 (UMCC 2981)**, LFA7, LFA8, LFA9, LFA10	630	400–230
MB	**LMB1 (UMCC 2982)**, LMB5	630	400–230
	LMB2, LMB3, LMB7, **LMB9 (UMCC 2984)**, LMB10	880	320–230–180–150
IMPB	LIB1, LIB4, **LIB7 (UMCC 2986)**, LIB10	880	320–230–180–150
	LIB2, LIB3, **LIB5 (UMCC 2985)**, LIB6, LIB8, LIB9	880	400–230
FINB	**LFB1 (UMCC 2987)**, LFB2, LFB3, LFB4, LFB6, **LFB10 (UMCC 2988)**	880	320–230–180–150
	LFB5, LFB7, LFB8, LFB9	630	400–230

* The strains sequenced are shown in bold and in brackets are indicated the correspondent UMCC code.

**Table 4:** PCR amplification of the 16S-23S rDNA intergenic spacer region (ISR).

Sample	Strain *	16S-23S rDNA ISR Size Fragments (bp)
MA	**BMA2 (UMCC 2989)**, **BMA8 (UMCC 2990)**, **BMA10 (UMCC 2991)**	600–700–800
IMPA	**BIA2 (UMCC 2992)**, BIA3, BIA5, **BIA6 (UMCC 2993)**, **BIA7 (UMCC 2994)**, BIA8, BIA9, **BIA10 (UMCC 2995)**	600–800
FINA	**BFA1 (UMCC 2996)**, **BFA2 (UMCC 2997)**	600–800
MB	**BMB5 (UMCC 2998)**, **BMB7 (UMCC 2999)**	600–700–800
IMPB	**BIB7 (UMCC 3002)**, BIB8	600–800
FINB	BFB3, **BFB6 (UMCC 3004)**, **BFB8 (UMCC 3006)**	600–800

* The strains sequenced are shown in bold and in brackets are indicated the correspondent UMCC code.

**Table 5:** Relative volatile organic compound (VOC) composition of sourdough and dough samples.

Chemical Compounds	Samples *					
	MA	MB	ImpA	ImpB	FinA	FinB
Acetaldehyde	2.22 ± 0.11	1.62 ± 0.13	8.43 ± 0.19	3.28 ± 0.01	8.31 ± 0.10	6.27 ± 0.05
2-Butenal	-	-	2.44 ± 0.16	1.56 ± 0.23	8.61 ± 0.65	3.80 ± 0.26
Hexanal	4.13 ± 0.17	3.86 ± 0.37	-	-	-	-
Heptenal	0.31 ± 0.04	0.66 ± 0.06	-	-	-	-
Benzaldehyde	-	-	0.55 ± 0.06	1.96 ± 0.12	0.86 ± 0.21	1.37 ± 0.05
Phenylacetaldehyde	-	-	6.80 ±0.47	7.62 ± 0.18	5.52 ± 0.33	6.08 ± 0.03
*Aldehydes*	*6.34*	*6.14*	*18.22*	*14.43*	*23.30*	*17.52*
Ethyl Acetate	39.73 ± 1.51	20.78 ± 0.43	7.17 ± 0.45	5.43 ± 0.23	4.08 ± 0.04	5.85 ± 0.15
Ethyl butanoate	-	-	-	-	0.86 ± 0.12	0.76 ± 0.13
Isoamyl acetate	2.03 ± 0.14	2.17 ± 0.23	-	-	-	0.39 ± 0.12
Ethyl hexanoate	-	0.38 ± 0.07	0.31 ± 0.00	0.20 ± 0.03	0.32 ± 0.02	0.64 ± 0.07
Hexyl acetate	-	0.26±0.01	-	-	-	-
Ethyl lactate	1.44 ± 0.04	2.14 ± 0.01	-	-	-	-
*Esters*	*43.19*	*25.74*	*7.48*	*5.64*	*5.26*	*7.26*
Ethyl alcohol	31.19 ± 0.22	44.05 ± 0.00	50.77 ± 0.66	53.19 ± 2.50	41.09 ± 2.65	40.51 ± 1.46
Isobutanol	-	-	1.40 ± 0.17	1.75 ± 0.11	-	2.51 ± 0.05
Isoamyl alcohol	4.30 ± 0.25	6.80 ± 0.32	6.42 ± 0.56	8.92 ± 1.06	6.70 ± 1.32	7.77 ± 0.25
1-Hexanol	1.96 ± 0.15	3.48 ± 0.42	1.60 ± 0.23	0.95 ± 0.12	1.12 ± 0.01	1.25 ± 0.06
2-Hexanol	-	-	0.64 ± 0.11	-	-	-
1-Octen-3-ol	0.30 ± 0.03	0.69 ± 0.03	-	-	-	-
Phenethyl alcohol	0.51 ± 0.01	3.27 ± 0.45	2.70 ± 0.38	7.44 ± 1.09	3.90 ± 0.62	8.22 ± 1.96
*Alcohols*	*38.27*	*58.30*	*63.52*	*72.26*	*52.82*	*60.25*
Acetic acid	5.34 ± 1.44	5.93 ± 0.09	1.23 ± 0.05	1.19 ± 0.09	1.11 ± 0.37	2.76 ± 0.35
Hexanoic acid	0.43 ± 0.07	0.94 ± 0.04	-	-	0.45 ± 0.12	0.31 ± 0.01
*Acids*	*5.76*	*6.87*	*1.23*	*1.19*	*1.11*	*2.76*
Limonene	2.49 ± 0.07	0.74 ± 0.01	1.08 ± 0.24	0.75 ± 0.11	0.96 ± 0.20	0.67 ± 0.06
Acetoin	-	-	7.42 ± 0.37	5.05 ± 0.33	9.39 ± 0.26	7.64 ±0.91
3,3-dimethyl hexane	-	-	-	-	2.48 ± 0.36	1.47 ± 0.19
1-butyl-2-propyl cyclopentane	-	-	-	-	3.24 ± 0.67	1.00 ± 0.40
2-Pentylfuran	0.92 ± 0.00	0.72 ± 0.02	-	-	-	-
Benzene,1,3-bis(1,1-dimethylethyl)	3.02 ± 0.28	1.03 ± 0.14	0.38 ± 0.06	0.69 ± 0.12	1.44 ± 0.02	1.44 ± 0.04
Others	6.43	2.49	8.89	6.49	17.51	12.21

* Data are expressed as % GC area ± standard deviation. The chemical classes of VOCs and their corresponding amounts are indicated in italics.

## Data Availability

Not applicable.
